# YKL-40 levels are associated with disease severity and prognosis of viral pneumonia, but not available in bacterial pneumonia in children

**DOI:** 10.1186/s12887-018-1345-y

**Published:** 2018-12-04

**Authors:** Xingge Yang, Guangyao Sheng

**Affiliations:** Pediatrics, the First Affiliated Hospital of Zhengzhou University, No. 1 East Jianshe Road, Zhengzhou, 450052 Henan China

**Keywords:** YKL-40, Children pneumonia, Biomarker

## Abstract

**Background:**

Viral pneumonia is the main type of community-acquired pneumonia (CAP) in children. YKL-40, a chitinase-like protein, is regarded as a biomarker of the degree of inflammation.

**Methods:**

Children who were diagnosed with CAP, including viral pneumonia, bacterial pneumonia, and dual infection, were included in the cohort study. The pathogenic diagnosis depended on PCR and immunoassay test. YKL-40 levels were examined twice by enzyme-linked immunoassay (ELISA).

**Results:**

Serum YKL-40 levels were higher in patients with pneumonia than in healthy controls. The admission levels of YKL-40 in serum and Bronchoalveolar lavage (BALFs) indicated a positive correlation with the serum levels of C-reactive protein and other inflammatory cytokines (IL-6 and TNF-α). The disease severity have no correlation with the admission serum levels of YKL-40. Meanwhile, reductions in YKL-40 levels from initial admission levels to day 5 post-admission were correlated with disease severity. The multiple logistic analysis indicated the decreased extent of serum YKL-40 level as an independent prognostic predictor of severe cases in patients with viral pneumonia.

**Conclusions:**

Reductions in serum YKL-40 levels on day 5 after receiving therapy is a possible prognostic biomarker for children with viral pneumonia.

## Background

Worldwide, 450 million patients are diagnosed with pneumonia, and approximately 4 million people die from this illness each year [1–3]. It is estimated that 156 million cases in children occur annually, and of these, 151 million occur in developing countries such as China [1]. Viral pneumonia is the most common community-acquired pneumonia (CAP) and a cause of lung infection in children. Approximately, 100 million cases occur every year in children, which accounts for the majority of patients with viral pneumonia [2]. The prognosis depends on various risk factors, including age, immune status, co-infection with bacterial pathogens and the type of pathogenic virus [4, 5]. The first evaluation as recommended by WHO paper cited by the authors should rely on clinical signs and symptoms, the requirement of supplemental oxygen administration and or ventilatory support, still lacking a rapid and safe method to ensure a diagnosis, exception made for bronchoalveolar lavage or lung biopsy [6].

But the study about the biomarker for evaluation of disease severity and prognosis is still infrequent, which lead to that pediatrician failed to precisely estimate the prognosis. Thus more intensive therapy including admission to the intensive care unit (ICU) more positively and preventive use of antibiotic drugs are not performed. Therefore, it is crucial for clinicians to investigate the biomarkers applicable for the prediction and assessment of prognosis.

YKL-40 is a chitinase-like protein and regarded as a pro-inflammatory cytokine mainly secreted by macrophages, which are involved in the inflammatory response and tissue injury [7, 8]. Hsiang-Ling Wang et al. have revealed that the prognostic value in a cohort of adult CAP and the plasma level of YKL-40 is positively correlated with the severity of CAP depicted by pneumonia severity index (PSI) and CURB-65 score [9]. YKL-40 has been recognized as a prognostic biomarker for ILD (interstitial lung disease) [10, 11]. Its role as a predictor of outcome in hypersensitivity pneumonia (HP) has also been proved in a recent HP cohort study [12]. But its role in predicting the prognosis of children with pneumonia, especially those with viral pneumonia, is not known.

The aim of present aim is to prove the clinical utility value of YKL-40 in BALF in children with pneumonia. Our study suggest that a clinician could evaluate their patient’s prognosis with two consecutive measurements of their YKL-40 serum levels, immediately after admission and day 5 after therapy initiation.

## Materials and method

### Study subjects and design

Children who were diagnosed with CAP, including viral pneumonia, bacterial pneumonia, and dual infection, were consecutively enrolled and their demographic and clinical data were retrospectively analyzed. The age of all children enrolled in the retrospectively study is ranging from 0.8 to 9.6. The mean age was 2.3 years old. The serum levels of YKL-40 in admission and at 5th day after therapy were measured. The clinical outcomes (including the incidence of entrance of ICU, mechanical ventilation and sepsis) and diseased severity was recorded. The correlation between admission levels of YKL-40 or decreased percentage after therapy and clinical prognosis in children with viral infection, bacterial infection and co-infection were analyzed and described respectively. The children who had a history of asthma, or immunodeficiency disease were excluded. The diagnosis of pneumonia was based on clinical characteristics, biochemical examination, cough, fever, and radiography. The diagnostic criteria of pneumonia included the presence of inflammatory exudation (alveolar or interstitial) by the chest X-ray with the simultaneous clinical manifestation of pulmonary infection, including fever, cough and difficult breathing. All patients diagnosed with pneumonia received the standard therapy according to the relevant guidelines for the CAP management in children and adult patients [13, 14].

All children or healthy volunteers enrolled in this study were in-patients at our institution from 2014 to 2017. The healthy volunteers included the out-patient children with no evidence of inflammation-related disease requiring a medical examination and collection of serum or blood samples. The retrospective observation study was approved by Medical ethic committee of the First Affiliated Hospital of Zhengzhou University. Informed consent was obtained from all study subjects.

### Pathogenic diagnosis

The sputum specimen was collected from children patients with pneumonia once the diagnosis was verified by chest X-ray. Gram stain, sputum culture were performed for the detection of specific bacterial strains including the *Pneumococcus* and *Haemophilus influenzae* as has been previously described [15]. The diagnosis of viral infection depended on the detection of the viral antigen by quantitative real-time PCR or multiplex RT-PCR following the standard protocols [16]. RNA was extracted from sputum specimens using Trizol (Invitrogen, USA). cDNA was synthesized by reverse transcription. The cyclic temperature settings were 95 °C, 30 s; 60 °C, 30 s; 65 °C, 30 s; amplified by 40 cycles with the last at 65 °C for 7 min. Human cytomegalovirus (CMV), adenovirus (AV) and parainfluenza virus(PIV) was assayed by fluorescent real-time PCR (BIO-RAD iCycler). For CMV detection, the forward and reverse primers were CMV-F: 5′-AACTGTACGTGCTGTGTGTACTAACTC-3′ and CMV-R: 5′-CTCGATAATGCGTTGTGCACCCCATAA-3′, respectively. For AV detection, the primers were AV-F: 5′-TGCGTAGTAGCCCTGGTGAA -3′; AV-R:5′-CATGTAGCGTGGTCGATGGTTC-3′; HPIVs-R: AGGTGACCGTGGGTCCACATG;HPIVs-F:ACTGTAGTAGGTTGTAGCTAG. The cyclic temperature settings were 95 °C, 30 s; 60 °C, 30 s; 70 °C, 30 s; amplified, 40 cycles.

### Definition of endpoint events and evaluation of diseased severity

The endpoint event for prognostic evaluation is the the incidence of sepsis secondary to pneumonia, use of mechanical ventilation and admission in Intensive Care Unit. The disease severity of the pneumonia was determined by the WHO recommended child pneumonia classification standard and based on clinical signs as previously mentioned, including the non-severe pneumonia, severe pneumonia and very severe pneumonia [17, 18]. To overcome the limitation of disease assessment by relying solely on the admission levels of YKL-40, we detected the levels of YLK-40 in the serum twice, immediately and on day 5 after admission. We calculated the reduction degree represented by the decreased percentage of YKL-40 levels on day 5 after receiving therapy and then ranked the reduction degree as follows: low group, ≤20%; median group, > 20% but ≤50%; and high group > 50%).

### YKL-40 and other inflammatory cytokine assays

BALF was collected via fiber optic bronchoscope as previously described [19]. The patient’s serum was collected twice, in admission (once the diagnosis was made immediately and prior to commencement of therapy) and on day 5 after admission. YKL-40 and other inflammatory cytokines, including C-reactive protein, interleukin (IL)-6, IL-10, and TNF-α, were examined in serum and BALF samples by enzyme-linked immunoassay (ELISA). The serum levels of YKL-40 were repeatedly measured respectively in admission and at 5th day after admission. The levels of BALF was measured only on admission.

### Mechanical ventilation

For children with severe pneumonia, the mechanical ventilation was applied according to synchronized intermittent mandatory ventilation (SIMV) combination with positive end expiratory pressure (PEEP). The tidal volume was kept in 10-12 ml/kg in these children. The weaning from mechanical ventilation was according to the improvement of blood oxygen saturation (FiO2 > 350) and normalization of respiratory rate (< 30 times /min) in autonomous respiration.

### Statistical analysis

Data were analyzed using SPSS software (SPSS, IL, USA) and were presented as the mean ± SD. The comparison between two groups was conducted by the Student t-test or Wilcoxon’s rank test for continuous variables and the Chi-squared or Fisher’s exact test for categorical variables. ANOVA was used for the multiple-group comparison. To overcome the limitation of disease assessment by relying solely on the admission levels of YKL-40, we detected the levels of YLK-40 in the serum twice, immediately and on day 5 after admission. We calculated the reduction degree represented by the decreased percentage of YKL-40 levels on day 5 after receiving therapy and then ranked the reduction degree as follows: low group, ≤20%; median group, > 20% but ≤50%; and high group > 50%) for the Spearman’s correlation analysis between the reduction degree of serum levels of YKL-40 and diseased severity. Spearman’s correlation coefficient was obtained for correlation analysis. Univariate and multiple logistic regression analyses were used to analyze the prognostic factors. A *p*-value of < 0.05 was considered as statistically significant.

## Results

### Characteristics of study subjects

Total of 321 subjects were enrolled in this study, including 50 healthy volunteers. The demographic characteristics and laboratory data of the study subjects are shown in Table 1. Regarding the age of the study subjects, no significant differences were found among the groups, including the sole viral, bacterial, and dual infection groups. The disease severity was divided into three classes according to the World Health Organization classification of pneumonia. At baseline, none of the subjects were receiving treatment with corticosteroids; they were all enrolled at the time of diagnosis before receiving therapy. The infection by *Pneumococcus* was detected in the 145 children and the infection by *Haemophilus influenzae* was detected in 67 children with pneumonia by sputum examination including direct smearing and sputum culture. The AV, CMV and HPIVs infection was found in 35, 45, 63 children respectively, which indicated that the HPIVs is the major viral pathogen in children viral pneumonia in our institution.

**Table 1:** Baseline demographic and clinical characteristics of study participants

Characteristics	Healthy controls (*N* = 50)	Viral Pneumonia (*N* = 104)	Bacterial Pneumonia (*N* = 110)	Co-infection (*N* = 66)	*P* value^b^
Age (years)	2.3(0.8–9.6)	1.8(0.2–8.9)	2.2(1.2–12.1)	2.4(0.5–9.7)	0.38
Gender (male, %)	34(68)	66(63)	52(47)	39(59)	0.31
Serum YLK-40 (ng/ml)	6.30 ± 2.27	18.48 ± 4.63	19.38 ± 3.34	19.32 ± 2.87	< 0.001
TNF-α (pg/ml)	NA	12.4 ± 7.85	13.6 ± 9.28	14.5 ± 10.23	0.24
IL-6 (pg/ml)	NA	32.3 ± 11.25	35.6 ± 8.17	39.5 ± 12.1	0.26
hsCRP (mg/dl)	NA	161.38 ± 34.78	158.25 ± 26.90	170.93 ± 29.36	0.34
IL-10 (pg/ml)	NA	83.96 ± 14.89	95.36 ± 8.98	84.36 ± 16.98	0.07
BALFs YLK-40 (ng/ml,n)	NA	26.45 ± 3.65(18)	34.87 ± 5.42(34)	33.63 ± 2.50(21)	0.02
Severity					
Not Severe (%)	NA	55.7	47.3	51.5	0.08
Severe (%)	NA	24.6	32.7	11.2	0.12
Very Severe (%)	NA	19.7	30.0	27.3	0.13
PO_2_ (mmHg)	NA	98.1 ± 1.0	97.2 ± 2.5	97.0 ± 3.6	0.41
SO_2_ (mmHg)	NA	95.5 ± 1.8	94.5 ± 1.3	94.9 ± 0.9	0.55
WBC (10^9^/L)	NA	9.8 ± 4.3	13.9 ± 3.5	14.2 ± 4.4	0.008
PCT (ng/ml)	NA	0.65 ± 0.26	1.33 ± 0.49	1.59 ± 0.87	0.02
Glucocorticoid use^a^, n(%) a	NA	20(19.2)	14(12.8)	12(18.2)	0.43

Abbreviations: *BALF* Bronchoalveolar Lavage Fluids, *CRP* high sensitive C-reactive protein, *WBC* white blood cell, *PCT* procalcitonin

^a^ Values at the period of hospitalization before colleting the serum sample at the second time

^b^*P* value < 0.05 was considered significant

### Admission YKL-40 levels in serum and BALFs of children with CAP

**Fig. 1:**
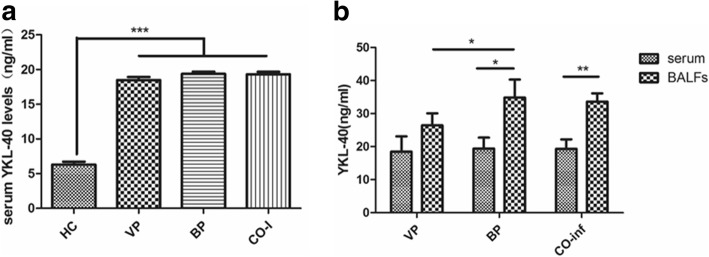
The admission serum and BALFs levels of YKL-40 in healthy control and child pneumonia. **a**. The serum levels of YKL-40 in healthy volunteers and different kinds of infectious pneumonia of children. VP, viral pneumonia. BP, bacterial pneumonia. CO-I, co-infection. **b**. The difference between the serum and BALFs levels as well as the BALFs levels among the VP, BP and co-infection. All data presented by mean ± SD. *** *P* < 0.001 **P < 0.01 *P < 0.05

Compared with the healthy controls, serum levels of YKL-40 in children with pneumonia was significantly higher (Fig. 1a). To verify the type of pneumonia was distinguished by YKL-40 levels in the serum or BALF, we compared the levels of YKL-40 in serum and BALF samples in the viral pneumonia and bacterial pneumonia groups (Fig. 1b). We observed no significant difference between the admission levels of YKL-40 in the serum of patients with viral pneumonia, bacterial pneumonia, or co-infection. But the levels of YKL-40 in the BALF specimens of patients with bacterial pneumonia were however significantly higher than those with sole viral pneumonia (*P* = 0.012), and the levels of YKL-40 in the BALF specimens were statistically higher than in the serum specimens from the same individuals (*P* = 0.007). However, in the sole viral pneumonia group, increased levels of YKL-40 in the BALF specimens as compared with the serum specimens was not observed.

### The correlation between admission levels of YKL-40 and other inflammatory biomarkers

**Fig. 2:**
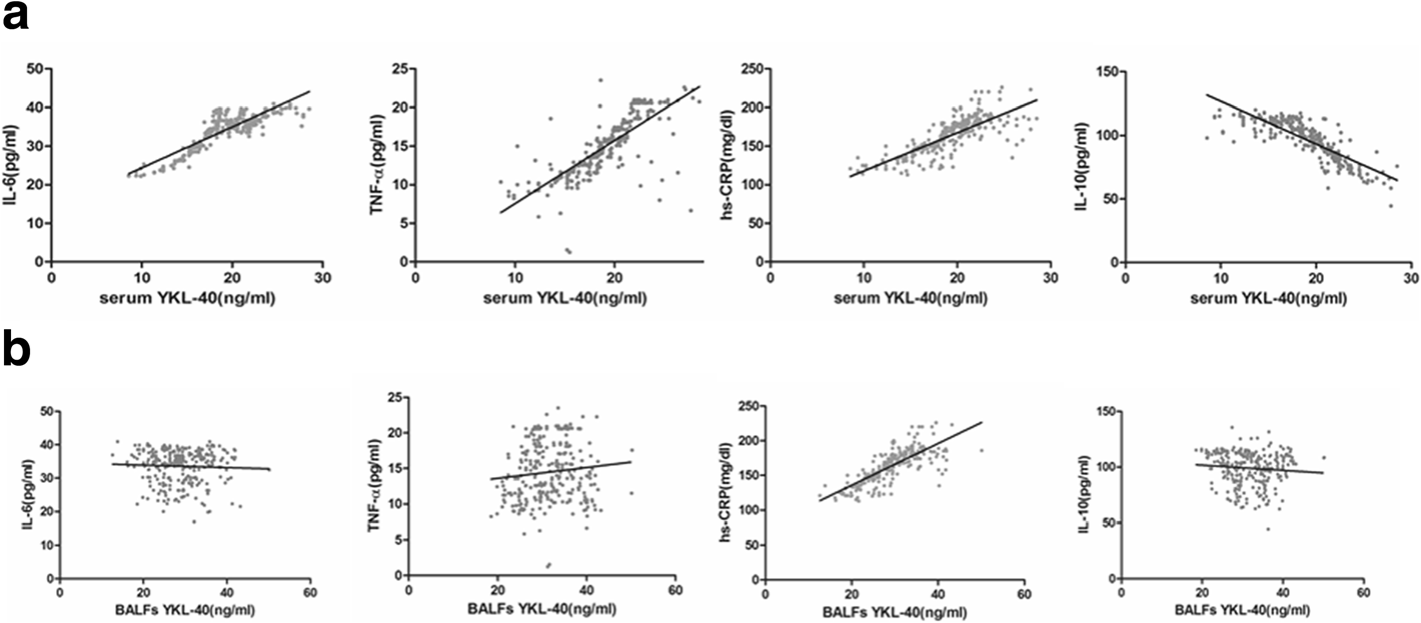
The linear correlation analysis between YKL-40 levels and classic inflammatory cytokine. **a**. The correlation between serum levels of YKL-40 and proinflammatory cytokine in circulating, including respectively pro-inflammatory IL-6 (*r*^*2*^ = 0.73, *P* < 0.0001), TNF-α (*r*^*2*^ = 0.58, *P* < 0001), hs-CRP (*r*^*2*^ = 0.61, *P* < 0.0001) and anti-inflammation IL-10 (*r*^*2*^ = 0.67, *P* < 0.0001). **b**. The correlation between BALFs levels of YKL-40 and proinflammatory cytokine in circulating, respectively in IL-6 (*r*^*2*^ = 0.0019, *P* = 0.46), TNF-α (*r*^*2*^ = 0.011, *P* = 0.0785), hs-CRP (*r*^*2*^ = 0.084, *P* < 0.0001) and IL-10 (*r*^*2*^ = 0.0079, *P* = 0.14). The slight correlation only emerged in between the circulating hs-CRP levels and BALFs levels. The data represented by mean ± SD, and *P* < 0,05 defined as significantly statistic difference

The levels of IL-6, TNF-α, and C-reactive protein were positively correlated with the serum levels of YKL-40, but the IL-10 levels were negatively correlated with YKL-40 levels in all 3 pneumonia subgroups (Fig. 2a). These results provide evidence that the YKL-40 is a pro-inflammatory cytokine accelerating tissue injury and might have an adverse effect on tissue repair and inflammation resolution. The levels of YKL-40 in the BALF specimens were also slightly positively correlated with the serum levels of C-reactive protein but not with the levels of IL-6 and TNF-α in all study subjects (Fig. 2b).

### The correlation between admission serum levels of YKL-40 and the disease severity of viral pneumonia

**Fig. 3:**
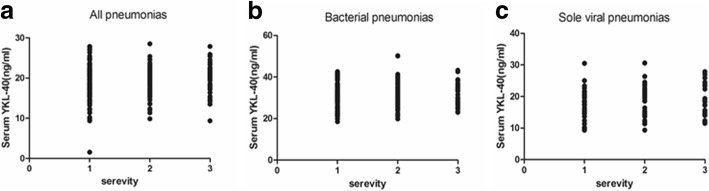
The spearman correlation analysis between diseased severity and serum levels of YKL-40. **a**. The spearman correlation between severity and serum levels in all child pneumonia patients. No significant correlation was observed (spearman *r* = 0.066, *P* = 0.28). **b**. The correlation between severity and serum levels in children patients with bacterial infection (spearman *r* = 0.16, *P* = 0.09). **c**. The correlation between severity and serum levels in sole viral pneumonia patients (spearman *r* = − 0.19, *P* = 0.12). No significant correlation between admission levels and severity in all children with viral or bacterial pneumonia. X axis represents the severity of childhood pneumonia. Non-severe pneumonia = 1, severe pneumonia = 2, very severe pneumonia = 3

The correlation between the admission serum levels of YKL-40 and severity of pneumonia was analyzed using the Spearman’s correlation coefficient; no significant correlation was observed (Fig. 3a, b and c). We further performed the Spearman’s correlation analysis in the different subgroups, and no obvious correlation was shown between the serum levels and disease severity, regardless of the type of pneumonia.

### The reduction degree of YKL-40 serum levels after standard therapy serves as an independent risk factor of viral pneumonia in children

**Fig. 4:**
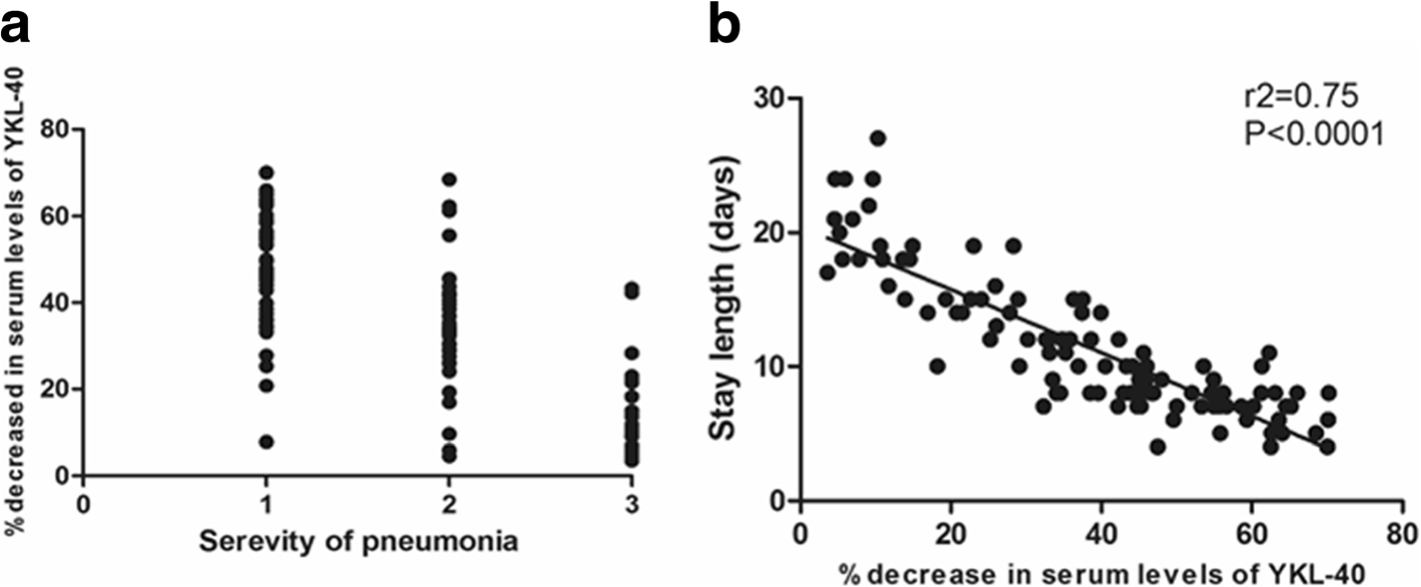
The correlation between decreased percentage of serum levels of YKL-40 after 5th days and diseased severity. **a**. The correlation analysis between severity class and decreased percentage (spearman *r* = − 0.68, *P* < 0.0001). The negative correlation was verified by the spearman correlation analysis. X axis represents the severity of childhood pneumonia. Non-severe pneumonia = 1, severe pneumonia = 2, very severe pneumonia = 3. **b**. The correlation analysis between severity class and median length of hospital stay (*r*^*2*^ = 0.75, *P* < 0.0001). The hospitalization stays period was negatively correlated with decreased extent after admission. *P* < 0.05 defined as significant statistic difference

Previous results have demonstrated that the admission levels of YKL-40 do not correlate with the disease severity. To determine a correlation with the severity of pneumonia, a univariate correlation analysis was performed. We found a negative correlation between the reduction degree of YLK-40 levels for the severity of disease and the median length of hospital stay (Fig. 4a and b) in the viral pneumonia group, but not in the bacterial pneumonia or co-infection groups (data not shown). In other words, the higher the percentage reduction in YKL-40 levels indicated the shorter hospital stay in the children with viral pneumonia. The logistic regression analysis identified the independent prognosis factors that were associated with the mechanical ventilation and ICU admission rates. The factors evaluated in the multivariate logistics regression model included age, history of previous pneumonia, procalcitonin levels, degree reduction of YKL-40 levels, and infectious type (Table 2). The independent risk factors for mechanical ventilation were identified as the age, infectious type, history of previous pneumonia, and reduction degree of YKL-40 levels. The independent risk factors for ICU admission were identified as the age, infectious type, and degree reduction of YKL-40 levels. For sepsis onset, the reduction degree of YKL-40 levels was the only independent risk factor.

**Table 2:** Logistics regression analysis for the factors associated with the prognosis of pneumonia

	OR	95% CI	*P* value
Rate of sepsis: Yes (45, 16.1%), No (235, 83.9%)			
Age (continuous variable)	1.21	0.85,1.54	0.652
Previous pneumonia(binary variable)	0.92	0.85,1.24	0.096
Decreased extent of YKL-40 (classified variable)	2.84	2.16,3.65	0.008
Infectious type (unordered variable)	1.23	0.97,1.48	0.078
Procalcitonin (continuous variable)	1.12	1.03,1.22	0.044
Mechanical ventilation rate: Yes (31, 11.1%), No (249, 88.9%)			
Age (continuous variable)	1.235	1.156,1.366	0.023
Previous pneumonia (binary variable)	2.02	1.68,2.26	0.034
Decreased extent of YKL-40 (classified variable)	1.69	1.26,2.08	0.012
Infectious type (unordered variable)	1.63	1.21,2.27	0.028
Procalcitonin (continuous variable)	1.021	0.916,1.148	0.189
Intensive Care Unit admission rate: Yes (36, 12.9%), No (244, 87.1%)			
Age (continuous variable)	1.23	1.12,1.51	0.041
Previous pneumonia (binary variable)	1.21	0.93,1.87	0.267
Decreased extent of YKL-40 (classified variable)	3.21	2.68,4.79	0.016
Infectious type (unordered variable)	1.35	1.21,1.45	0.030
Procalcitonin (continuous variable)	1.09	0.91,1.17	0.526

Data are presented as hazard ratios, representing the relative risk of adverse therapeutic events and poor prognosis. The independent risk factors of mechanical ventilation including the age, infectious type, infections history in the previous and decreased extent of YKL-40. The age, infectious type, decreased extent of YKL-40 not associated with the increased rate of mechanical ventilation but also considered as the independent risk factors of ICU admission

## Discussion

The present study revealed the usefulness of YKL-40 in the prognostic assessment of child pneumonia at first, especially in viral pneumonia. Although we could not demonstrate a positive correlation between serum level of YKL-40 and disease severity, the degree of reduction of YKL-40 levels on day 5 after admission could be considered as a prognostic biomarker and might guide the clinical decisions in children with pneumonia (e.g., need more intensive therapy and care, including mechanical ventilation or admission to ICU).

YKL-40, a recently discovered protein, is involved in airway inflammation, a potential biomarker of asthma, and a member of the chitinase and chitinase-like protein family. It is secreted in the airway mucosa by the inflammatory cells that reside in the bronchial mucosal and airway epithelial cells [20–22]. Chitinase might contribute to the inflammation and bronchial remolding in T helper 2 (Th2)-airway inflammation, an immune response mediated condition. It has been reported that YKL-40 levels were increased in children with asthma and were correlated with the markers of disease severity [21]. The prognosis of hypersensitivity pneumonitis is also correlated with the serum levels of YKL-40 [12]. The admission level of YKL-40 in the serum could predict disease progression and estimate mortality risk in patients with hypersensitivity pneumonitis. Meanwhile, in a cohorts of adults with CAP, the serum level was correlated with the pneumonia severity index, CURB-65 scores, length of hospital stay, and APACHE-II scores, which suggested that the levels of YKL-40 might have the potential to guide the treatment of CAP in adults [8].

We found that the higher admission level of YKL-40 in children with pneumonia than healthy volunteers. And the increased YKL-40 levels in BALFs compared to the serum levels was observed in the children with bacterial infection. In children with sole viral infection, the significant difference between the serum levels and BALFs levels of YKL-40, which might be attributed to the infiltrates being confined to within the pulmonary interstitial lesion in most cases of viral pneumonia. But it did not predict the disease severity of pneumonia in children, which differs for the finding in adults. A previous study found that immune dysfunction was associated with increased disease severity in infants [23, 24]. This suppression of the adaptive immune response also has an adverse influence on the prognosis in septic children [25]. Therefore, the fact that there is no correlation between admission levels of YKL-40 and diseased severity might be attributed to the YKL-40 levels, which are decreased in the immunocompromised status of the severe cases in children, relative to the mild and slight cases [26]. In addition, the results are different from the finding in adults with CAP as mentioned in the introduction section. Therefore the admission levels of circulating YKL-40 have little correlation with the severity of pneumonia, which might be due to the immune suppression status for severe infectious pneumonia in children.

We noticed that the YKL-40 levels of BALF specimens were higher than the peripheral circulation in bacterial pneumonia, which support the fact suggestion that YKL-40 is secreted by the locally infiltrated inflammatory cells. To verify the correlation between the serum levels of classical pro-inflammatory cytokines and the BALF levels of YLK-40, we performed a linear correlation analysis. The levels of YKL-40 in the BALF specimens is positively correlated with the serum levels of CRP, which suggested that the level of YKL-40 in BALF specimens represented only the activity of inflammation at the lesion location rather than the systemic inflammation due to the endotoxin release or inflammation cascade. However, the difficulty in obtaining BALF samples limits its utilization for the repeated detection of admission levels of YKL-40 for monitoring diseased status. In a previous study associated with cystic fibrosis, Fantino et al. revealed that only the airway local levels of YKL-40 reflected the activity in lung tissue for the infant and young children with early cystic fibrosis [27]. The results above demonstrated that YKL-40, secreted by neutrophils, often serves as a confined inflammatory cytokine and is enriched in local inflammatory tissue rather than being released into the peripheral circulation. Therefore, it also could partly explain why the admission levels of YKL-40 were not correlated with the disease severity of pneumonia.

Eventually, to illustrate the predictive value in the evaluation of reduction degree serum levels of YKL-40 in children viral pneumonia, we applied the multivariable logistic regression analysis to explore the independent risk factors that might be correlated with prognosis. We found the reduction degree of YKL-40 is negative correlated with the admission of ICU, incidence of mechanical ventilation and sepsis. The results demonstrated that the reduction degree of YKL-40 levels, serving as a pro-inflammatory cytokine, was associated with the prognosis of child pneumonia, including the median length of stay, sepsis rate, mechanical ventilation rate, and ICU admission rate.

### Study limitation

In the study, there were still several study limitations that should be taken into account in further and in-depth investigation. At first, we could not collect the BALF specimen because of the difficulty in the taking samples although the fact that the secretion of YKL-40 were produced by local inflammatory cells resident in respiratory tract. Secondly, the predictive value of degree of reduction in serum levels YKL-40 after 5 days compared than the admission levels was only available in the children with sole viral pneumonia, which might limit the more comprehensive application of this biomarker in clinical practice.

## Conclusions

In conclusion, the degree of reduction of YKL-40 in the serum has a potential value in the prognostic assessment and prediction of disease severity. The YKL-40 levels in BALF specimens might reflect inflammatory activity at the lesion location and disease severity. Our study indicated that dynamically monitoring the levels of this inflammatory cytokine has potential value in the assessment of infectious diseases in children. More prospective clinical trials are needed to verify our finding.

## Abbreviations

BALF: Bronchoalveolar lavage
CAP: Community-acquired pneumonia
ELISA: Enzyme-linked immunoassay
HP: Hypersensitivity pneumonia
ICU: Intensive care unit
IL: Interleukin
ILD: Interstitial lung disease
PSI: Pneumonia severity index
Th2: T helper 2
